# Chronic critical illness: reason for concern during and after ICU admission!

**DOI:** 10.1186/cc14674

**Published:** 2015-09-28

**Authors:** Carlos Augusto R Feijó, Allison EPP Borges, Eduardo Q da Cunha, Francisco A de Meneses, Marina P Albuquerque, Natália LP Aragão, Tamara O Pinheiro, Túlio S de Aguiar

**Affiliations:** 1 General Hospital of Fortaleza, Papicu, Fortaleza, Ceará, Brazil

## Introduction

Evolution of healthcare assistance in the ICU, due to its technological apparatus, has increased survival of critically ill patients substantially. In this ballast, patients who require prolonged length of stay, as well as demand continued intensive care support (particularly mechanical ventilation (MV)), are increasingly identified. These patients suffer from chronic critical illness (CCI), a rapidly growing syndrome which has immune, neuroendocrine and metabolic particularities-today, they represent 5-15% of all patients admitted to the ICU.

## Objective

To describe the epidemiological characteristics of CCI in an adult ICU and to analyze possible predicting factors of evolution to CCI at admission to the ICU.

## Methods

Retrospective analysis of patients admitted to the General Hospital of Fortaleza-SESA from November 2014 to February 2015. The CCI was defined as prolonged MV (>21 days, for at least 6 hours per day). Descriptive statistical analysis was utilized for demographic characters, *t *test for evaluation of continuous variables and chi-square test for categorical variables.

## Results

From 86 patients admitted in that period, 13 (15%) were identified as chronically critical. The mean age of these patients was 57 ± 22 years, and 61% of them were female, mean SOFA score (at admission) was 6.5 ± 3.3 points and mean APACHE II score was 18 ± 5.5 points, statistically similar to the other patients. At admission, 61.3% had hypertension, 46.1% were diabetics; chronic kidney disease (CKD) and chronic cardiovascular disease were identified in 23% each. The presence of diabetes mellitus (DM) and CKD were significantly higher in the CCI population (*p *= 0.048 and *p *= 0.033, respectively). The mean MV time, ICU and hospital length of stay were significantly increased in the CCI population, with 34 ± 12 days (p < 0.001).

## Conclusion

The prevalence of CCI in this study follows the current literature. High SOFA and APACHE II scores do not seem able to identify the patients who will evolve from severely ill to CCI. The present study suggests that comorbidities, at admission, may indicate which patients will survive acute critical illness to become CCI-as well as CKD and DM. The significant post-ICU mortality highlights the need for more suitable follow-up for the CCI population after discharge.

